# Effects of acceptance of disability on death or dialysis in chronic kidney disease patients: a 3-year prospective cohort study

**DOI:** 10.1186/s12882-015-0197-z

**Published:** 2015-12-05

**Authors:** Hsin-Hung Chiang, Hanoch Livneh, How-Ran Guo, Mei-Ling Yen, Tzung-Yi Tsai

**Affiliations:** Department of Nursing, Dalin Tzuchi Hospital, The Buddhist Tzuchi Medical Foundation, Chiayi, Taiwan; College of Nursing, Chang Gung University of Science and Technology, Chiayi, Taiwan; Rehabilitation Counseling Program, Portland State University, Oregon, USA; Department of Environmental and Occupational Health, College of Medicine, National Cheng Kung University, Tainan, Taiwan; Department of Occupational and Environmental Medicine, National Cheng Kung University Hospital, Tainan, Taiwan; Department of Medical Research, Dalin Tzuchi Hospital, The Buddhist Tzuchi Medical Foundation, No. 2, Minsheng Rd., Dalin Township, Chiayi, County 62247 ROC Taiwan; Department of Nursing, Tzu Chi University of Science and Technology, Hualien, Taiwan

**Keywords:** Acceptance of disability, Chronic kidney disease, Dialysis, Mortality, Taiwan

## Abstract

**Background:**

Acceptance of disability (AOD) is a useful construct that assesses the ability of a patient to psychologically cope with chronic diseases, but its effect on long-term outcomes of patients with chronic kidney disease (CKD) remains unclear. This study aimed to evaluate the relation between AOD level and clinical outcomes in a cohort of CKD patients in Taiwan.

**Methods:**

262 CKD patients without dialysis at a hospital in Taiwan were consecutively recruited, from 2010 to 2011, and followed up for 3 years. At enrollment, demographic and clinical data were obtained, including baseline level measurement of AOD, using the Acceptance of Disability Scale-Revised (AODS-R). During follow-up, the authors assessed the effect of AOD on progression to dialysis and all-cause mortality by using Cox proportional hazard regression analysis.

**Results:**

Of the patients included in the analyses, 145 (55.3 %) whose total scores of AOD were below the median (86.00) were regarded as having low AOD at enrollment. At the end of 3-year follow-up, 25 have died and 57 initiated dialysis. Participants with low AOD were more likely to have the composite end-point of progression to dialysis or death (adjusted hazard ratios [AHR] = 1.89, 95 % confidence interval [CI]: 1.18-3.20). In addition, CKD stage at IV or above and hemoglobin level were found to be associated with the occurrence of the composite end-point.

**Conclusion:**

AOD was associated with an increased risk for poor clinical outcomes, thus suggesting that prompt awareness and management of the psychological reactions may improve clinical outcomes of patients with CKD.

## Background

Chronic kidney disease (CKD) has become a global public health burden over the past decades [1]. Due to the irreversible nature of CKD, most patients gradually progress to end-stage renal disease (ESRD) and require dialysis or renal *transplantation*. The global ESRD prevalence is estimated to continue to rise at an annual rate of 7 % and exceed 2 million by 2010 [2]. In Taiwan, where the prevalence is among the highest in the world, ESRD has put a substantial burden on healthcare resources. A governmental report in 2012 revealed that while Taiwanese ESRD patients constituted less than 0.3 % of the population, they took up about 7 % of the total healthcare budgets, surpassing that of cancer patients [3].

In addition to the enormous economic impact, CKD is also a significant public health burden. A cohort study in Taiwan of 462,293 subjects reported that nearly 10.3 % of deaths were due to CKD, and that 40 % of CKD deaths occurred in patients younger than 65 years-old [4]. Therefore, CKD patients typically experience many symptoms over a prolonged period of time and are frequently diagnosed with psychiatric disorders, such as depression. A meta-analysis showed that depression was common in patients with CKD, with a pooled prevalence of 21 %, approximately two- to four-fold higher than that in the general population [5]. Furthermore, CKD patients with depressive symptoms have over three-fold increased risk of death relative to those without the symptoms [6], suggesting that the improvement of psychological adjustment would prolong the survival rate.

In general, a psychological intervention program for patients with chronic diseases appears to help them to accept their disabilities, learn to better cope and live with their diseases, and adapt to the ensuing physical and psychosocial sequelae [7]. Acceptance of disability (AOD) is considered a key factor that accounts for this improvement in psychosocial adjustment [8, 9]. AOD is associated with viewing one’s disability as a manageable barrier that does not devalue life or minimize one's remaining abilities, and this can be achieved through personal coping efforts and the realization that one can successfully overcome many of life’s restrictions and limitations [10]. To specifically measure AOD, Linkowski developed an AOD scale based on concepts inherent in the notion of acceptance of loss [11]. Numerous studies have used this scale to examine disease and disability acceptance among patients with chronic conditions such as Ehlers-Danlos syndrome [12], diabetes [13], spinal cord injury [8], and stroke [14]. Overall, these results indicated that the higher the level of disability acceptance, the more successful is the coping with the condition and the better is the perceived quality of life of the patients. Nonetheless, the long-term effects of AOD on clinical outcomes in patients with chronic diseases remain unclear. To fill this gap, we conducted a study to examine the relationships between AOD and two clinical outcomes, namely, progression to dialysis and all-cause mortality, among CKD patients in Taiwan.

## Methods

### Study design and population

We recruited consecutive CKD patients from either outpatient or inpatient departments, located at a hospital in southern Taiwan, from January 2010 to December 2011. The eligibility criteria at baseline included: (1) age of at least 20 years; (2) no physician-based diagnosis of alcohol abuse, dementia, psychosis, or depression; (3) ability to express opinions in Mandarin or Taiwanese; (4) diagnosis of CKD by a physician, but not yet undergoing dialysis; and (5) willingness to consent to a blood test on the survey date. Before enrollment, all participants received detailed written and verbal information regarding the aims and protocol of the study and signed informed consents. To ensure participants’ anonymity, all questionnaires were marked with encryption code to facilitate data analysis, but lacking any personal identifiers. This study was approved by the institutional review board of the Dalin Tzuchi Hospital, Buddhist Tzuchi Medical Foundation, Taiwan (No. B10201011).

### Measures

#### Assessment of AOD level

AOD was assessed by the revised version of the AOD scale (AODS-R), developed by Groomes and Linkowski [9]. It is a 32-item questionnaire that was developed as a brief multidimensional self-reporting instrument for the assessment of four domains: enlargement of scope of values, subordination of physique, containment of disability effects, and transformation from comparative to asset values. Each item is rated on a 4-point Likert scale, ranging from 1 (strongly disagree) to 4 (strongly agree). Thus, the AODS-R total score ranges from 32 to 128, where higher scores indicate a higher level of disability acceptance. Ten of the items address positive values. The remaining 22 items address negative values. A principal component analysis extracted four factors corresponding to the psychometric characteristics of the original version, thus providing support for the conceptual validity of the AODS-R [9]. Previous studies have demonstrated that the AODS-R has good internal consistency in different groups of patients with chronic illnesses and disabilities [14–16]. The data collected in our study yielded a Cronbach’s α of 0.92 for the scale.

#### Covariates

Patient interviews and reviews of medical records were performed to collect the information on demographic and clinical characteristics at enrollment. The demographic data considered included sex, age, marital status, educational level, job status, living status, religious beliefs, monthly income, and certain lifestyle factors, such as use of alcohol and tobacco, exercise habits, and presence of sleep disturbances. Alcohol consumption was recorded as “less than 2 times per week” or “at least two times per week”; smoking status was recorded as “non-smoker” or “current or ex-smoker.” Those who exercised regularly (that is, weekly) were regarded as having “exercise habits”. Sleep disturbance was defined as waking up at night more than twice without external factors during the week before the interview. Clinical characteristics included the presence of a chronic disease (*i.e.* at least one of the following: diabetes mellitus, hypertension, heart disease, or stroke), CKD stage, hemoglobin level, and serum albumin level.

#### Primary clinical outcomes

The primary clinical outcomes comprised death (all-cause mortality) and progression to dialysis. The date of initiation of dialysis was determined during monthly face-to-face interviews at outpatient clinics or by telephone. If the researchers suspected that a patient has died, they contacted the participant's family directly for confirmation, and to obtain the date of death. All participants were followed from the date of enrollment until death or the end of 3-year follow-up, whichever came first.

### Statistical analysis

After using the median of the range of obtained AOD scores as the cut-off, we split participants into two groups: “high” and “low” AOD levels. The mean, standard deviation (SD), and percentage of demographic and disease data between the two AOD groups were then compared employing two-sample t tests for continuous variables and chi square tests for categorical variables. Next, incidence rates of adverse events were presented as the number of cases per 1000 person-months (PMs). Finally, we applied the Cox proportional hazard model to compute the AHR and its 95 % CI for primary outcomes associated with a low AOD level. Assumptions of the proportional hazard models were confirmed by using log-log plots and cumulative Schoenfeld residual plots. All inferential analyses were two-tailed using an alpha level of 0.05, and all statistical analyses were performed using SAS version 9.3 (SAS Institute Inc., Cary, NC, USA).

## Results

### Basic characteristics of participants

Of the 267 consecutive qualified CKD patients, 3 refused to participate in the follow-up survey and 2 were excluded because primary outcomes occurred within the first month of follow-up, leaving insufficient time for the establishment of temporal relationship. Therefore, data on 262 patients were included in the final analysis. The mean age of patients was 64.3 years, and 61.5 % were men (Table 1). The mean AOD score was 85.02 (± 5.36), and 55.3 % (145/262) of the participants scored at a low AOD level (at AOD score below the median of 86.00).

**Table 1:** Demographic and clinical characteristics of all CKD patients with low and high AOD

Variables	All subjects (*n* = 262)	Low AOD (*n* = 145)	High AOD (*n* = 117)	*p*
Demographic characteristics				
Age (years)	64.26 ± 13.25	64.08 ± 12.14	65.29 ± 14.25	0.47
Gender				
Male	161 (61.5)	83 (57.2)	78 (66.7)	0.12
Female	101 (38.5)	62 (42.8)	39 (33.3)	
Educational level				
High(≥12^th^ grade)	66 (25.2)	45 (31.0)	51 (43.6)	0.04*
Low(<12^th^ grade)	196 (74.8)	100 (69.0)	66 (65.4)	
Marital status				
Single	18 (6.9)	12 (8.3)	6 (5.1)	0.32
Married	244 (93.1)	133 (91.7)	111 (94.9)	
Job status				
Employed	73 (27.9)	43 (29.7)	30 (25.6)	0.47
Unemployed	189 (72.1)	102 (70.3)	87 (74.4)	
Living status				
Alone	22 (8.4)	12 (8.3)	10 (8.6)	0.92
Cohabitating	239 (91.6)	133 (91.7)	106 (91.4)	
Monthly income^★^				
≥ NT$30,001	50 (19.1)	27 (18.6)	23 (19.7)	0.83
≤ NT$30,000	212 (80.9)	118 (81.4)	94 (80.3)	
Religious beliefs				
Yes	223 (85.1)	123 (84.8)	100 (85.5)	0.88
No	39 (14.9)	22 (15.2)	17 (14.5)	
Alcohol drinking				
Low (≤ 1 times/week)	247 (94.3)	136 (93.8)	111 (94.9)	0.71
High (≥ 2 times/week)	15 (5.7)	9 (6.2)	6 (5.1)	
Cigarette smoking				
Yes	107 (40.8)	59 (40.7)	48 (41.0)	0.95
No	155 (59.2)	86 (59.3)	69 (59.9)	
Regular exercise				
Yes	171 (65.3)	85 (58.6)	86 (73.5)	0.01*
No	91 (34.7)	60 (41.4)	31 (26.5)	
Sleep disturbance				
Yes	166 (63.4)	117 (80.7)	79 (67.5)	0.02*
No	96 (36.6)	28 (19.3)	38 (32.5)	
Clinical characteristics				
CKD stage				
Stage I, II, or III	104 (39.7)	49 (33.8)	55 (47.0)	0.03*
Stage IV or V	158 (60.3)	96 (66.2)	62 (53.0)	
Other chronic diseases				
≤ 1	90 (34.4)	42 (29.0)	48 (41.0)	0.04*
≥ 2	172 (65.6)	103 (71.0)	69 (59.0)	
Hemoglobin (g/dL)	11.12 ± 2.04	10.86 ± 2.11	11.43 ± 1.89	0.02*
Serum creatine (mg/dL)	4.97 ± 1.58	5.13 ± 5.76	4.77 ± 5.97	0.62
Serum albumin (g/dL)	3.51 ± 0.51	3.46 ± 0.57	3.57 ± 0.41	0.07

Abbreviation: AOD, Acceptance of disability

Data is presented as mean ± SD or n (%)

^★^US $1 = 30.23 NTD at the study time period

*p < 0.05

Patients with a low AOD level were more likely to have a low educational level (*p* = 0.04), did not exercise regularly (*p* = 0.01), had sleep disturbance (*p* = 0.02), were diagnosed with CKD stage IV or above (*p* = 0.03), had concomitant chronic diseases (*p* = 0.04), and had a low hemoglobin level (*p* = 0.02) (Table 1).

### Incidence and hazard ratios for death and initiation of dialysis

Overall, the mean follow-up was 31.17 months, and 82 patients (31.3 %) either died (*n* = 25, 9.5 %) or progressed to dialysis (*n* = 57, 21.8 %). CKD patients with low AOD were more likely to progress to dialysis (27.6 % *vs.* 14.5 %; *p* = 0.03) and die (13.1 % *vs.* 5.1 %; *p* = 0.01). After adjustment for the significant factors in the univariate analysis, we found that lower AOD was associated with an elevated but non-significant risk of death (AHR = 1.82; 95 % CI: 0.90–4.90). In contrast, AOD level was associated with a statistically significant risk of progression to dialysis (AHR = 1.95; 95 % CI: 1.04–3.34) (Table 2).

**Table 2:** Effect of AOD on risk of death or progression to dialysis in CKD patients

	Event (n)		Incidence**		AHR^†^(95 % CI)
	Low AOD	High AOD	Low AOD	High AOD	
Death	19	6	4.51	1.49	1.82 (0.90-4.90)
Dialysis	40	17	9.43	4.33	1.95 (1.04-3.34)

Abbreviations: AOD, Acceptance of disability; AHR, adjusted hazard ratios

**Incidence is presented as cases per 1000 person-months

^†^Adjusted for the educational level, exercise habit, CKD stage, sleep disturbance, chronic disease, and hemoglobin level

### Multivariable analysis of factors associated with the composite end-point

When we combined death and progression to dialysis as a composite end-point, we found that low AOD score was associated with an increased risk (AHR = 1.89; 95 % CI: 1.18–3.20) after adjustment for potential confounding factors. The other independent risk factors included CKD stage IV or above (AHR = 1.82; 95 % CI: 1.21–4.21), and a low hemoglobin level (AHR = 0.81; 95 % CI: 0.70–0.92) (Table 3).

**Table 3:** Multivariable analysis of factors associated with the composite endpoint of death and progression to dialysis

Variables	CHR (95 % CI)	AHR^†^(95 % CI)
AOD		
Low	2.34 (1.51-3.49)*	1.89 (1.18-3.20)*
High	-	-
Educational level		
Low	0.98 (0.49-1.38)	1.01 (0.58-1.71)
High	-	-
Other chronic diseases		
≤ 1	1.34 (0.82-2.17)	1.31 (0.81-2.13)
≥ 2	-	-
Regular exercise		
No	0.89 (0.41-1.57)	0.97 (0.50-1.44)
Yes	-	-
Sleep disturbance		
Yes	1.17 (0.74-2.84)	1.04 (0.94-2.98)
No	-	-
CKD stage		
Stage IV or V	2.26 (1.70-5.36)*	1.82 (1.21-4.21)*
Stage I, II, or III	-	-
Hemoglobin (g/dL)	0.78 (0.63-0.88)*	0.81 (0.70-0.92)*

Abbreviations: AOD, Acceptance of disability; CHR, crude hazard ratios; AHR, adjusted hazard ratios

^†^Adjusted for the other variables in this table

*p < 0.05

## Discussion

The present 3-year longitudinal follow-up study of CKD patients who were not yet on dialysis showed that those with low AOD had an increased risk for progression to dialysis, which is consistent with findings of previous studies that CKD patients who experience negative emotions tend to also experience worse clinical manifestations [5, 17]. A study by Fischer and colleagues [18] also found that depressive symptoms predicted a 15 % greater risk of progression to ESRD among patients with CKD, but this increased risk did not reach statistical significance. This discrepancy in findings could be partially attributed to difference in potential confounders measured in both analyses. It could also be attributed to different definitions of disease stage between the two studies since Fischer et al. [18] directly used the value of glomerular filtration rate (GFR) to evaluate the disease severity of CKD, and not classification of stage.

There are at least several potential reasons for the association between low AOD and poor clinical outcomes. First, negative emotions, such as depression, are known to be associated with treatment non-adherence [19] and unhealthy lifestyle such as malnutrition and physical inactivity that may accelerate the decline in serum albumin and thus progression of CKD [20, 21]. Systematic inflammation induced by a distressed mood may be another plausible explanation; many studies demonstrated that CKD patients with depressive symptoms had higher levels of circulating inflammatory markers, such as interlukin-6 (IL-6), tumor necrosis factor-α (TNF-α), and C-reactive protein (CRP), all of which play important roles in the pathogenesis of cardiovascular diseases and worsen the prognosis of CKD [5, 22]. Third, negative mood influences the sensitivity to glucocorticoid hormones and leads to immunosuppression through activation of the hypothalamic-pituitary-adrenal (HPA) axis [23], which substantially increases the risk of acute infection and mortality [24]. Therefore, the implementation of standardized psychosocial assessment and of patient care procedures, in routine care, may help early referral of CKD patients for further therapeutic interventions that could delay the progression of disease.

The findings of our study revealed that CKD patients with stage IV and above had a higher risk of poor clinical outcomes, and this is compatible with a large population-based study showing that late-stage CKD was a significant risk factor for mortality among renal failure patients in Taiwan [25]. It could be argued that patients with advanced stage of kidney failure typically experience higher predisposition to cardiovascular diseases or renal-related co-morbidities, and consequently are at an elevated risk of death [26]. Additionally, the present study showed that a one-unit increase in hemoglobin was associated with a 19 % decrease in the risk for the composite end-point, and this is compatible with findings of a previous study [27]. Patients with decreased levels of hemoglobin are more prone to anemia, which can evoke left ventricular hypertrophy and cardiovascular mortality [28]. Therefore, subnormal hemoglobin levels in CKD patients should be recognized and managed at earlier stages of disease progression.

### Strengths and limitations

In contrast to the available data on the influence of psychological responses on clinical outcomes in renal disease patients who receive dialysis treatment, the findings from this 3-year prospective cohort study focused on CKD subjects who did not yet undergone dialysis. Our findings provide strong support for the importance of psychological factors, such as disability acceptance, in affecting clinical outcomes, thus supporting usefulness of therapeutic regimens geared at promoting psychosocial adjustment to CKD. Several limitations, nevertheless, should be considered when interpreting the results of the present study. First, all participants were drawn from a single hospital in southern Taiwan, so the results might not be generalizable to other CKD subjects, especially those from non-Chinese populations. However, previous research has also often been limited by such factors as subjects' ethnicity, geographical location, nationality and the nature of the medical data available, suggesting that this limitation is not unique to our study. Second, in this study, we only assessed AOD at the beginning of the follow-up time period. It is likely that AOD scores could change over time. However, failure to consider the effect of time-varying predictors on end-points would tend to underestimate, rather than overestimate, an association [29], so AOD is more likely to increase rather than decrease over time [14]. Therefore, this potential concern is not likely to influence the conclusions of our study. Third, we assessed sleep disturbance using a single-item question, so the psychometric soundness of such an assessment may be suspect and thus must be interpreted cautiously.

### Implications

The findings of this study have important implications for future research and clinical practice. Given that our data were drawn from a single hospital may restrict the generalizability of these findings. Accordingly, it is recommended, that larger community-based studies, with a comprehensive battery of psychological and clinical measures, be employed to identify undiagnosed patients in order to validate our findings. Furthermore, longitudinal measurement of disability acceptance should be implemented to verify if the present findings are consistent across the process of psychological adaptation to CKD. As far as clinical practice is concerned, the findings of this study indicate that AOD level could serve as an independent risk factor for poor clinical outcomes, suggesting that the AODS-R scale could be judiciously applied when assessing reactions towards life with a chronic illness, such as CKD. Finally, the present findings, especially if supported by future research, also point to the importance of routinely observing patients for negative psychological responses, and, if feasible, instituting appropriate interventions for routine clinical practice.

## Conclusion

We found that low AOD was independently associated with an increased risk of poor clinical outcomes. In addition, patients with CKD stage IV and above and those with low hemoglobin levels also had an elevated risk of poor clinical outcomes. These findings, together with previous results, provide further support that poor psychosocial adaptation, as assessed by low levels of disability acceptance, may have an imperative role in CKD prognosis. Therefore, it may be tentatively recommended that routine psychological screening, through the use of AOD at diagnosis of CKD, followed by monitoring and timely management of its progression, could be beneficial to patients with CKD.

## Abbreviations

AHR: Adjusted hazard ratios
AOD: Acceptance of disability
AODS-R: Acceptance of Disability Scale-Revised
CHR: Crude hazard ratios
CI: Confidence interval
CKD: chronic kidney disease
CRP: C-reactive protein
GFR: glomerular filtration rate
HPA: Hypothalamic-pituitary-adrenal
IL-6: Interleukin 6
PMs: Person-months
SD: Standard deviation
TDQ: Taiwan Depression Questionnaire
TNF-α: Tumor necrosis factor-α
